# Impact of Acetaldehyde Addition on the Sensory Perception of Syrah Red Wines

**DOI:** 10.3390/foods11121693

**Published:** 2022-06-09

**Authors:** Luca Garcia, Cédrine Perrin, Valérie Nolleau, Teddy Godet, Vincent Farines, François Garcia, Soline Caillé, Cédric Saucier

**Affiliations:** SPO, Université de Montpellier, INRAE, Institut Agro, 34000 Montpellier, France; luca.garcia@umontpellier.fr (L.G.); cedrine.perrin@inrae.fr (C.P.); valerie.nolleau@inrae.fr (V.N.); teddy.godet@inrae.fr (T.G.); vincent.farines@umontpellier.fr (V.F.); francois.garcia@umontpellier.fr (F.G.); soline.caille@inrae.fr (S.C.)

**Keywords:** acetaldehyde, red wine, Syrah, sensory analysis, polyphenol

## Abstract

Two experimental Syrah red wines with different polyphenol contents were used to study the impact of acetaldehyde addition on olfactory perception. Free acetaldehyde levels were measured in red wine by Head Space-Gas Chromatography-Mass Spectrometry (HS-GC-MS) to determine the acetaldehyde combination levels for those wines. Significant differences were observed for both sensory threshold and acetaldehyde combination for the wines. A descriptive sensory analysis of the wines was then performed by using a trained panel and a Hierarchical-Check-All-That-Apply (HCATA) analysis of the wines with or without acetaldehyde addition. The results show that classical cited sensory descriptors for acetaldehyde (overripe apple and oxidized apple) varied significantly between the control wines and those with acetaldehyde addition. Non-acetaldehyde related descriptors (fresh vegetable, fresh flowers, cocoa, and meat juice) were also significantly impacted in the samples with increasing acetaldehyde additions. This suggests possible interactions between acetaldehyde and other volatile compounds that can create antagonistic or synergistic effects between the molecules or at the olfactory receptor level.

## 1. Introduction

Acetaldehyde is a volatile molecule and one of the most abundant carbonyl compounds in many fermented foods [1,2]. Generally, it represents more than 90% of the total aldehyde content in wines [3]. Its main origin is alcoholic fermentation [4] where it plays an important role in yeast metabolism. It is formed by the decarboxylation of pyruvate and can subsequently be converted into ethanol by alcohol dehydrogenase enzymes [5]. It can also be formed during the whole wine process and especially during wine aging by the oxidation reaction of ethanol catalyzed by copper or ferrous ions [6,7]. 

Acetaldehyde is a very reactive carbonyl compound and can combine quickly and strongly with sulfur dioxide (SO_2_), a major wine antioxidant and antimicrobial molecule to form non-volatile adducts known as hydroxysulfonates [8,9]. These molecules represent the most important proportion of the bound form of acetaldehyde in wine [10].

In addition to that, acetaldehyde can react easily as an electrophile in acidic media and bind amino acids or peptides, such as glutathione [11]. It can also be involved in polymerization with phenolic compounds [12]. These reactions result in the formation of ethylidene bridges either between flavan-3-ol units [13], anthocyanin units [14], or flavan-3-ol and anthocyanin units [12]. Reactions between anthocyanins and acetaldehyde can lead to the formation of anthocyanin-derived pigments, such as pyranoanthocyanins [15,16]. These pigments contribute to the color evolution of red wines, from purple-red for a young wine to orange-red color for an aged wine.

Total acetaldehyde can be found in wines at a concentration ranging from 5 to 100 mg/L [2,17,18]. Higher concentrations can be found in fortified wines, such as sherry wines, from 90 up to 500 mg/L [17]. From a sensory point of view, acetaldehyde is often characterized by the term “oxidized” [19]. A recent study by Pelonnier-Magimel et al. [20] on the sensory characteristics of Bordeaux red wines produced without added sulfites showed that those wines had a much higher frequency of sensory defects. These defects were related to oxidation in a proportion close to 50%. At a low concentration, acetaldehyde may also enhance the fruity aroma [18] and intensify the appearance of the term “green apple” [21]. However, at higher levels, it is reminiscent of nuts and, at a very high concentration, it can generate the appearance of notes of bruised and rotten apple [18] or ripe apple [22]. At these high concentrations, above 100 mg/L, it is then considered as a defect [3]. In terms of mouth-feel properties, the reactions of condensation between acetaldehyde and phenolic compounds that occur during wine aging may impact the astringency of red wine [23]. 

Acetaldehyde interactions with other molecules can impact the free acetaldehyde concentration in wine and therefore impact its perception threshold. In the literature, different ranges of total acetaldehyde perception threshold can be found: 0.5 mg/L in hydroalcoholic solution [24], 30 mg/L in model wine solution [25] up until 100 and 125 mg/L in wine [18,26].

The sensory perception of a wine is the result of complex interactions between many volatile and non-volatile compounds [27]. These differences in perception can come from interactions between the compounds themselves, synergistic/antagonistic effects, and chemical and physiological phenomena [28]. The perception of acetaldehyde is then dependent on the matrix it is found.

The objectives of this article are:

- To determine the orthonasal perception threshold of acetaldehyde in two Syrah red wines with different polyphenol contents in order to study the effect of the red wine matrix on the perception of acetaldehyde.
- To measure the free acetaldehyde levels in the Syrah red wines to determine the acetaldehyde combination levels.
- To study the effect of increasing acetaldehyde addition on the sensory descriptors of Syrah red wines by Hierarchical-Check-All-That-Apply (HCATA) analysis.

## 2. Material and Methods

### 2.1. Reagents, Solvents, and Standards

Acetaldehyde (≥99%), methyl-2-methylbutyrate (99%), sodium chloride, phloroglucinol (≥99%), catechin (≥98%), and ascorbic acid were purchased from Sigma-Aldrich (Saint-Quentin Fallavier, France); acetaldehyde (food grade, ≥97%) and ammonium sulfate were obtained from Merck (Darmstadt, Germany). Oenin chloride was obtained from Extrasynthèse (Genay, France).

### 2.2. Wine Samples and Preparation

Two 100% Syrah red wines with different Total Polyphenol Index (TPI) [29] were obtained from Pech-Rouge Experimental Unit (INRAE, Gruissan, France):

- Syrah 1 (S1): TPI of 41.
- Syrah 2 (S2): TPI of 80.

Different amounts of acetaldehyde were spiked into the red wine samples. The acetaldehyde additions to the wine samples were performed the day before in a cold room (+6 °C) to prevent acetaldehyde evaporation during the sample preparation. After the addition, the samples were stored with the minimum head-space at 17 °C in a cellar for at least 8 h before sensory and chemical analysis, to reach free acetaldehyde equilibrium as reported by Arias-Pèrez et al. [30].

### 2.3. Chemical Analysis

#### 2.3.1. Oenological Parameters

Classical oenological parameters methods were measured following International Organisation of Vine and Wine (OIV) reference methods. Alcoholic percentage was determined by Fourier transformed infrared spectroscopy—FTIR (WineScan, FOSS France, Nanterre, France) (OIV-MA-BS-08) [31], free and total sulfur dioxide by the automated iodometric method (Titromatic, Crison Instruments, Alella, Spain (OIV-MA-AS323-04B) [32], and pH by the potentiometric method (pH meter Consort C3010, Consort bvba, Belgium) (OIV-MA-BS-13) [33]. Finally, the Total Polyphenol Index (TPI) was determined as absorbance at 280 nm using a UV mc2 spectrophotometer (Safas, Monaco) [29].

#### 2.3.2. Analysis of Free Acetaldehyde in Wines by HS-GC-MS

Free acetaldehyde was determined by head-space gas chromatography coupled to a quadrupole mass spectrometer detector (HS-GC-MS) following the method described in Carrascon et al. [34] with slight modifications. GC-MS analysis was carried out on a GC Trace Ultra gas chromatograph (Thermo Fisher, Waltham, MA, USA) and coupled to a ISQ Series mass spectrometer (Thermo Fisher, Waltham, MA, USA). A DB-WAX (30 m × 0.25 mm × 0.25 µm) capillary column (Agilent Technologies, Santa Clara, CA, USA) was used for the chromatographic separation. For the analysis, 5 mL of sample, 1 g of sodium chloride, and 20 µL of methyl-2-methylbutyrate ethanolic solution (1250 mg/L) as internal standard were added into a 10 mL headspace vial, and were incubated at 40 °C for 15 min. After this, 400 µL of the headspace was injected into a PTV injector, working in split mode (1:7 split ratio) and kept at 200 °C. An AOC-5000 autosampler (Shimadzu, Kyoto, Japan) with a static headspace unit was used and the 1 mL gas-tight syringe was heated at 50 °C. After the injection, the hot syringe was cleaned by purging for 5 min with nitrogen (Air Products, Allentown, PA, USA). The oven started at 50 °C for 4 min and then was raised to 220 °C at 50 °C/min and was kept at this temperature for 5 min. The carrier gas employed was helium (Air Products, Allentown, PA, USA) at a constant flow of 1.5 mL/min. GC-MS transfer line temperature was 240 °C, ion source temperature was 200 °C and quadrupole 150 °C. Spectra were acquired using electron impact ionization (EI, 70 eV) in single ion monitoring (SIM) mode. The *m*/*z* used for quantification were 29 for acetaldehyde and 88 for methyl-2-methylbutyrate.

External calibration curves in model wine (5 g/L tartaric acid, 13% ethanol, and pH 3.6) containing known amounts of acetaldehyde were prepared.

#### 2.3.3. Determination of Phenolic Composition

##### Anthocyanins

The quantification of monomeric anthocyanins was performed as described previously with slight modifications [35]. The wines were filtered with 0.45 µm PTFE filter (Macherey-Nagel, Düren, Germany) and directly injected (7.5 µL) on a UPLC system Waters Acquity (Saint-Quentin-en-Yvelines, France) with a photodiode array detector (PDA). A reversed-phase UPLC Acquity UPLC BEH C18 column (50 mm length, 2.1 mm internal diameter, 1.7 µm particle size) from Waters was used for chromatographic separation. The autosampler was kept at 8 °C. The method used a binary gradient with mobile phase A containing 0.1% (*v*/*v*) aqueous trifluoroacetic acid and mobile phase B containing acetonitrile. The column temperature was set at 50 °C. The 40 min elution method at flow of 0.25 mL/min was 1% B (0 min), 1–8.8% (0–5 min) B, 8.8–20.6% (5–30 min) B, 20.6–96% (30–30.5 min) B, isocratic with 96% B (30.5–34 min), 96–1% (34–34.1 min) B, and isocratic with 1% B (34.1–40 min). The detection was monitored at 520 nm. 

An external malvidin-3-O-glucoside calibration curve was used. The results are expressed as mg malvidin-3-O-glucoside equivalent (M3G eq.)/L. The analyses were performed in triplicate.

##### Flavanols

The flavanol composition of the wines was studied by phloroglucinolysis reaction (acid-catalyzed depolymerization in the presence of a nucleophilic agent) following the procedure described by Carrascon et al. [36] with slight modifications. A total of 400 μL of wine sample was evaporated to dryness in a centrifugal solvent evaporator (Genevac, Ipswich, UK). The pellet was dissolved in 600 μL of a solution of 50 g/L of phloroglucinol and 10 g/L of ascorbic acid in methanol-HCl 0.2 N. The mixture was heated at 50 °C for 20 min to complete the reaction, then cooled in an ice bath, and finally, 600 μL ammonium acetate (200 mM) was added to stop the reaction. Samples were centrifuged at 12,000 rpm at 5 °C for 10 min and the supernatants were collected for analysis. The conditions of the chromatographic apparatus are the same as those mentioned in Section 2.3.3 Flavanols part. 

The method used a binary gradient with mobile phase A containing 0.1% (*v*/*v*) aqueous trifluoroacetic acid and mobile phase B containing acetonitrile. The 22 min elution method at flow of 0.45 mL/min was 2% B (0 min), 2–6% B (0–10 min), 6–20% B (10–16 min), 20–99% B (16–16.1 min), isocratic with 99% B (16.1–18 min), 99–2% B (18–18.1 min), and isocratic with 2% B (18.1–18 min). The column temperature was 40 °C. Eluting peaks were monitored at 280 nm. This analysis allows us to have access to the nature and relative proportions of the terminal and extension subunits. 

An External catechin calibration curve was used, and quantification was conducted in equivalents of catechin and concentration of epicatechin, epigallocatechin and epicatechin-3-O-gallate, and their phloroglucinol adducts were estimated using their response factors relative to catechin [37]. The analyses were performed in triplicate.

### 2.4. Sensory Analysis

#### 2.4.1. Participants

The panel was composed of 13 panelists (3 men and 10 women, average age of 55 years), selected on the basis of their sensory performances and interest [38] and trained in the descriptive sensory analysis of wines. For this study, the judges were informed about the addition of a chemical element to wines and signed a consent form before the sensory experiments.

#### 2.4.2. Orthonasal Thresholds (Ot) for Acetaldehyde

Orthonasal thresholds for acetaldehyde were defined in S1 and S2. Six wine samples with acetaldehyde concentrations of 5, 15, 20, 25, 30, 35, and 40 mg/L were prepared. The concentration range for acetaldehyde was established around the orthonasal detection limits of acetaldehyde found in the scientific literature [25,30,39].

The method adopted consisted of a succession of triangular tests comparing the wine without addition to the wines with acetaldehyde addition, by increasing concentrations. The analysis was repeated in a second independent session. 

The analysis was conducted in individual testing booths, and the temperature of the tasting room was 21 °C. Samples (4 cl) were served at 17 °C, in black wine glasses in order to avoid any potential visual influence on the participant evaluations, with three-digit random codes, different for each glass. Each glass was covered with a lid to protect the samples from aroma evaporation, particularly concerning acetaldehyde.

Scores were collected by a computerized data acquisition system (FIZZ software, Biosystèmes, Couternon, France).

A logistic regression (1) on the percentage of correct answers as a function of concentration (Re p) was used to calculate the threshold value of 50% of correct answers:

(1) Re p=11+exp[−(a+b×conc)]

where Re p the probability of correct answers, conc the concentration of acetaldehyde, and a and b are the parameters of the model.

Data analysis was performed using XLSTAT software (Addinsoft, Paris, France) [40,41]. 

#### 2.4.3. HCATA Methodology 

The aim of this part of the study was to characterize the olfactory perceptions of S1 and S2 according to acetaldehyde concentration. A range of 10 acetaldehyde concentrations (0, 5, 15, 25, 40, 55, 75, 85, 100, and 120 mg/L) was analyzed using the hierarchical CATA methodology [42]. This method allows a more “natural” hierarchical structure of attributes and reduces cognitive effort. Concentrations were chosen to respect the acetaldehyde level naturally present in red wines, ranging from 5 to 100 mg/L with an average of 30 mg/L.

Prior to this, judges attended two training sessions to exercise understanding and consistently use attributes and also to familiarize themselves with the methodology.

Olfactory standards were prepared by adding compounds to red Syrah wine and were adopted to help the judges to identify and remember the sensory attributes (Table S1. Supplementary data).

The analyses were conducted in individual testing booths; the temperature of the tasting room was 21 °C. The samples were evaluated in a monadic service, with the same order of presentation for all judges, in ascending order of acetaldehyde concentration. This experimental protocol was chosen to analyze the olfactory changes progressively induced by acetaldehyde addition. The evaluation was carried out in duplicate in two sessions. The samples were served at 17 °C, in black wine glass covered with a lid, identified with three-digit random codes. 

For each sample, the judges had to choose between 1 and 6 most pertinent attributes, from a list of 61 olfactory terms (Table 1). The attributes were classified hierarchically into 9 families and 20 sub-families.

Scores were collected by a computerized data acquisition system (FIZZ software, Biosystèmes, Couternon, France).

An average reproducibility index (Ri) (2) was calculated to assess the individual performance of each judge:

(2) $$R_{i}=\left(1/n\right)\times \sum \left[2\times {\mathrm{des}}_{\mathrm{rep}}/\left({\mathrm{des}}_{\mathrm{rep}1}+{\mathrm{des}}_{\mathrm{rep}2}\right)\right]$$

where des_rep_ is the number of same terms used by the judge for each replicate, des_rep1_ and des_rep2_ are the total numbers of terms used by the judge for the first and second replicates (respectively), and n is the number of concentrations duplicated. This parameter, ranging from 0 to 1, was used in previous works [43].

The data obtained were binary and their analysis was performed using XLSTAT software (Addinsoft, Paris, France). In order to analyze the significant differences between the acetaldehyde concentrations for each attribute, a Cochran’s Q test was performed. 

The correspondence analysis (on the chi2 distance) was performed on the contingency table. Only attributes with a *p*-value less than 0.3 were considered in order to limit noise.

## 3. Results and Discussion

### 3.1. Chemical Analysis of the Syrah Red Wines 

#### 3.1.1. Chemical Characterization of the Red Wines

According to Table 2, the two red wines had significantly different polyphenol content. Concerning the Total Polyphenols Index, the difference was very important (twice), respectively, 41 and 80 for S1 and S2 wines. These values were in accordance with anthocyanin and flavanol contents (Tables S2 and S3. Supplementary Information) as the concentrations of these compounds for S2 measured twice as high than those of S1.

The percentage of alcohol and pH values were very close for both samples. The values of free SO_2_ were different but remained low (8 and 14 mg/L for S1 and S2, respectively).

#### 3.1.2. Quantitative Study of the Free Acetaldehyde of the Syrah Red Wines with Increasing Acetaldehyde Addition 

Due to the high reactivity of acetaldehyde, the levels of free acetaldehyde in the wine samples used for the sensory analysis were analytically controlled by Head-Space-Gas Chromatography-Mass Spectrometry in order to calculate the real free acetaldehyde concentration in the samples after 8 h of equilibration. For the wine samples prepared for the orthonasal threshold (OT), the concentration of total acetaldehyde added to wine ranged between 0 and 40 mg/L and, for the HCATA, the spiked concentration ranged between 0 and 120 mg/L. For these concentrations, a difference of free acetaldehyde concentration was found and was different for the two wines (Table 3). 

**Figure 1:**
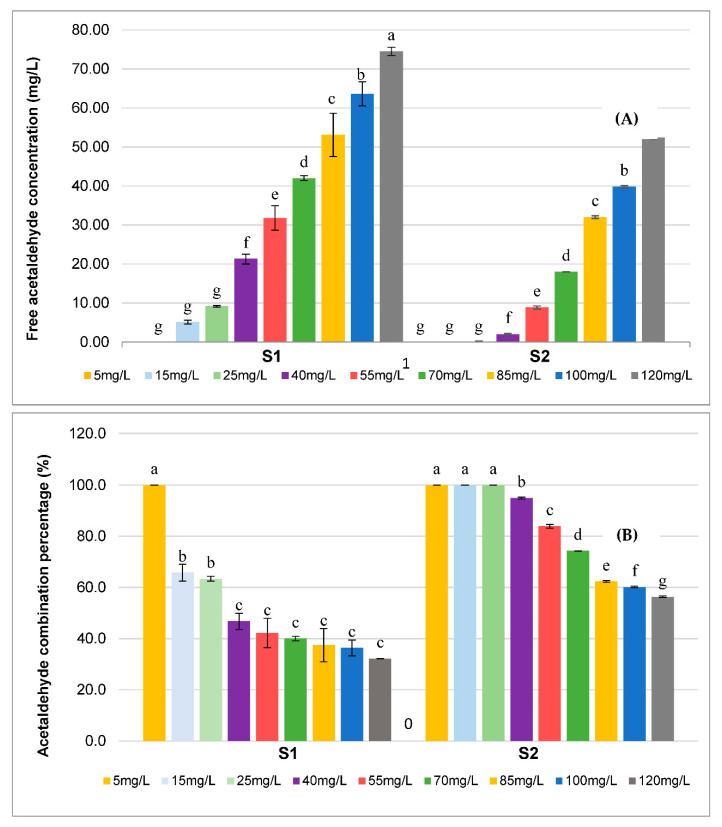
Evolution of free acetaldehyde concentration (**A**) and the corresponding combination percentages for HCATA samples (**B**). Different letters indicate significant differences between samples according to Tukey’s test (*p* < 0.05).

Acetaldehyde is very reactive with free SO_2_ [10], so the differences in concentrations could induce variations in the free acetaldehyde concentrations. However, the measured values of free SO_2_ were very close for both samples. Theoretically, 30 mg/L of SO_2_ could bind 20 mg/L of acetaldehyde in wine [44]. Therefore, the amount of free SO_2_ in the S1 wine could approximately bind only 5 mg/L of acetaldehyde and only 9 mg/L in S2. The difference in concentration of free acetaldehyde (Figure 1A) can then not be explained by the combination between SO_2_ and acetaldehyde (Figure 1B).

Once SO_2_ has been combined, the unreacted fraction of acetaldehyde may form ethyl bridges with polyphenols. Bueno et al. [45] have shown that the proportion of free acetaldehyde was more important in a wine with a lower concentration of aldehyde-reactive polyphenols (ARPs), such as malvidin 3-O-glucoside or epigallocatechin. Given that the concentrations of monomeric anthocyanins and flavanols were twice as high in the S2 wine as S1 (Table 2), these important differences in polyphenol content can explain the variation of the combination percentage of spiked acetaldehyde between the S1 and S2 wine samples (Figure 1B). For S2, acetaldehyde was completely combined for concentrations below 40 mg/L, while for S1, it was only for concentrations below 5 mg/L.

These results highlight that polyphenol concentration impacted free acetaldehyde levels due to adduct formation. As these differences in free acetaldehyde concentration may have an impact on the sensory perception of the Syrah red wine samples, sensory experiments were performed and are reported in the next sections of this article.

### 3.2. Influence of Acetaldehyde Concentration on Syrah Sensory Threshold and Descriptors

#### 3.2.1. Orthonasal Threshold of Acetaldehyde

Acetaldehyde was added to the two different Syrah wines in the range from 5 to 40 mg/L. Threshold values were calculated for 50% of the correct answers (Table 4) using a logistic regression logit model for link function on the percentage of correct answers versus the concentration. 

For the S1 wine, the average perception threshold calculated was 6.9 ± 3.7 mg/L. This value was lower than that found in the literature for model and white wines [25,39]. However, as shown by Arias-Pérez et al. [30], free acetaldehyde concentration in a model red wine enriched in non-volatile wine compounds was much lower than the total acetaldehyde concentration added. The low perception thresholds for the S1 wine compared to the literature is mainly due to the fact that we are expressing our acetaldehyde as free acetaldehyde and not as added total concentration.

For the S2 wine samples, the orthonasal threshold could not be established as no-significant differences were detected by the panel up to 40 mg/L. This is in accordance with the free acetaldehyde concentration measured by HS-GC-MS after addition. Indeed, no free acetaldehyde was found in the sample for the 5 to 30 mg/L addition, but the sample spiked with 40 mg/L had a free acetaldehyde concentration of 0.7 mg/L (Table 3 and Table 4). The absence of free acetaldehyde for some samples indicates that the spiked acetaldehyde was completely in combined form and therefore not in the sensory active form. This difference in acetaldehyde combination between S1 and S2 (Figure 1) could be explained in part by a higher level of free SO_2_, but above all by a higher concentration of polyphenol in S2 than in S1 (Table 2). These results show that the matrix impacts the orthonasal perception threshold of acetaldehyde due to the formation of adducts with non-volatile compounds, such as polyphenols.

#### 3.2.2. HCATA Analysis of Panel Performance

The ranking of the reproducibility index (Ri) associated with a judge allowed to evaluate the accuracy of a judge’s performance (Table S4, Supplementary Information). In the present work, the maximum Ri value was found to be 0.53, which corresponds to 53% of the common terms between the two replicates for a judge. The minimum value found for a judge was 0.13 (the median value being 0.30). The performances of the jury were quite homogeneous, so all the results from all the judges were kept.

#### 3.2.3. HCATA Characterization of the Olfactory Sensory Properties of the Syrah Wines Containing Acetaldehyde 

The olfactory characterization of Syrah wines as a function of the amount of added acetaldehyde (5 to 120 mg/L) using the HCATA method showed that acetaldehyde increased or exacerbated various descriptors depending on its concentration for each of the two Syrah wines.

Cochran’s Q tests showed that there were significant differences at a risk α of 5% for four attributes for each of the two Syrah wines and a further five and eight attributes at a risk α of 10% (Table 5). 

The results show that the concentration of acetaldehyde in the Syrah red wines had an impact on their olfactory perception. Indeed, depending on the level of acetaldehyde, the frequency of citation of descriptors associated with Syrah wines varied significantly and in the same way that the attributes associated with the acetaldehyde molecule, such as “baked apple”, “overripe apple”, or “oxidized green apple”. 

**Figure 2:**
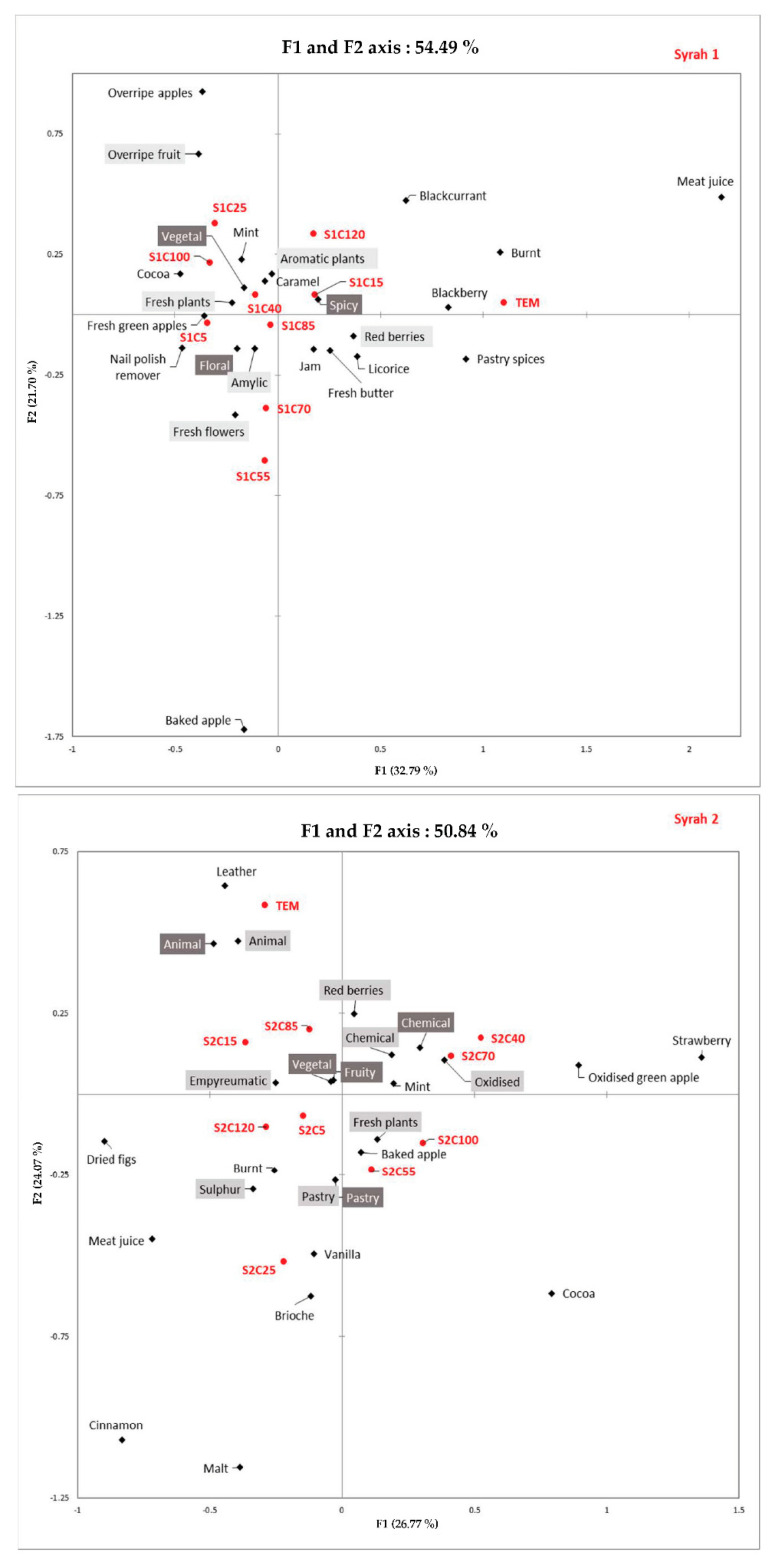
Correspondence analysis of HCATA results for each sample. SXCY with X = the number of samples and Y = the corresponding spiked concentration. Families–Sub-families–olfactory terms. TEM = control wine without acetaldehyde addition.

A correspondence analysis (CA) was then performed (Figure 2) for each Syrah wine. 

For S1, the first two axes represented 54.49% of the total variance. On the one hand, the control wine without acetaldehyde addition (TEM) was described, on axis 1, by the terms “meat juice”, “blackberry”, “burnt” as well as “spicy” and “pastry”. On the other hand, wine samples with the highest concentrations (100 and 120 mg/L) and with the concentration at 25 mg/L were characterized, on axis 2, by the descriptors “overripe fruit” and “overripe apple”. Wine samples with intermediate concentrations (55 and 70 mg/L) were described, on axis 2, by “fresh flowers” and “baked apple”.

The HCATA method showed that, for S1 wine, a low concentration of spiked acetaldehyde (15 mg/L) significantly increases the apparition of “red berries” notes.

Intermediate concentrations of acetaldehyde (55 and 70 mg/L) induced also the detection of the “baked apple” descriptor that is considered pleasant. whereas higher concentrations (100 and 120 mg/L) tended to be characterized by “overripe apple/overripe fruit”, corresponding to unpleasant notes. In addition to that, an increase in the number of citations for the descriptors “vegetable” and “fresh plants” and “fresh green apples” according to acetaldehyde concentration can be noted. These observations were in agreement with those in former studies [10,22], which showed that the descriptor “bruised and overripe apple” was associated with wines with a high acetaldehyde concentration. Moreover, Arias-Pérez et al. [30] demonstrated that a high concentration of acetaldehyde in a model red wine enhances the “vegetables” note. For S2, the correspondence analysis (Figure 2) showed that the first two axes represented 50.84% of the total variance. The control wine without acetaldehyde addition (TEM) was described, on axis 2, by the terms “leather” and “animal”.

On the contrary, wine samples with higher concentrations (70 and 100 mg/L) were characterized, on axis 1, by the descriptors “strawberry”, “oxidized”, and “oxidized green apple”. The wine sample with the 25 mg/L concentration was described, on axis 2, by “pastry”, “brioche”, “malt”, and “vanilla”. 

The HCATA results show that the amount of acetaldehyde influenced the olfactory perception of this second wine. Nevertheless, some descriptors highlighted for some concentrations of spiked acetaldehyde were not in accordance with the literature. It must be noted that the addition of acetaldehyde at low concentrations (5 and 15 mg/L) did not differentiate the samples from the control, but a concentration of 25 mg/L in the wine exacerbated “pastry” notes, such as “cinnamon” and “brioche”. Intermediate concentrations (40 and 70 mg/L) increased the intensity of the “oxidized green apple” notes, rather associated with higher concentrations [18]. Moreover, it must be noted that there was an increase in notes associated with “pastry” at higher levels of spiked acetaldehyde. Samples with a high concentration of acetaldehyde, such as 100 mg/L, were also characterized by significantly stronger “cocoa” notes. The sample spiked with 120 mg/L differed significantly with notes of “cinnamon” and “dry figs” that were more pronounced.

## 4. Conclusions

Overall, the results of this work confirm that acetaldehyde had an impact on the olfactory perception of Syrah red wines with different polyphenol contents. At high acetaldehyde concentrations, classical sensory descriptors, such as “overripe apple” or “oxidized apple”, were cited. At intermediate and low concentrations, other descriptors were identified, such as “vegetal”, “red berries”, “fresh flowers”, or “meat juice”. This suggests that acetaldehyde may interact with other volatile compounds to create antagonistic or synergistic effects between the molecules or at the olfactory receptor level. Its impact also differs depending on the red wine considered and the polyphenol content is an important parameter as shown by our results. Indeed, the rapid covalent or non-covalent interaction of acetaldehyde with polyphenols seemed to occur as evidenced by the very different perception threshold and free acetaldehyde measurement in our red wine samples. Further research is needed to better understand acetaldehyde–aromas interactions in red wine sensory characteristics and to understand its reactivity with polyphenol or other compounds during ageing.

## Figures and Tables

**Table 1:** List of families and 20 sub-families and olfactory terms used for the HCATA analysis.

Family	Subfamily	Descriptors
**Fruity**	Red berries	Blackberry, Blackcurrant, Raspberry, Strawberry
	Stewed fruit	Prune, Jam, Baked apple
	Dry fruit	Coconut, Hazelnut, Nut, Dried Fig
	Overripe fruit	Overripe apples
**Floral**	Fresh flowers	Violet, White flowers, Rose
	Dried flowers	Faded roses
**Vegetal**	Fresh plant	Grass, Fresh green apples, Peppers
	Dry plant	Tobacco, Black tea
	Undergrowth	Humus, Truffle, Mushroom
**Spicy**	Spicy	Licorice, Clove, Black pepper, Nutmeg
	Aromatic plants	Thyme, Laurel, Eucalyptus, Black olive, Mint, Anise
**Pastry**	Pastry	Vanilla, Cinnamon, Brioche, Biscuit, Pastry spices, Praline
	Yeast	Malt
**Animal**	Animal	Leather, Meat juice
**Lactic**	Lactic	Fresh butter, Rancid butter, Milk
**Empyreumatic**	Empyreumatic	Cocoa, Chocolate, Coffee, Smoked, Burnt, Toasted bread, Caramel
**Chemical**	Amylic	Candy, Banana
	Chemical	Nail polish remover, Varnish
	Oxidized	Oxidized green apple, Sweet wine
	Sulfur	Tar, Sulfur

**Table 2:** Classical oenological parameters and phenolic characterization of the different Syrah red wines. Values represent means of triplicate determination ± standard deviation. M3G eq: Malvidin-3-O-glucoside equivalent.

Samples	Grape Variety	Vintage	TPI	Ethanol% (*v*/*v*)	Free SO_2_ (mg/L)	Total SO_2_ (mg/L)	pH	Flavanols (g/L)	Anthocyanins (mg/L M3G eq)
**S1**	Syrah	2020	41	14.1	8	18	3.88	0.69 ± 0.013	223 ± 1
**S2**	Syrah	2020	80	14.6	14	25	3.95	1.28 ± 0.016	510 ± 4

**Table 3:** Free acetaldehyde concentrations measured by HS-GC-MS of the spiked Syrah red wine samples for OT (A) and HCATA analysis (B). Values represent means of triplicate determination ± standard deviation.

**A. Measured Free Acetaldehyde Concentrations of Spiked Syrah Red Wine Samples for OT**											
**Acetaldehyde addition (mg/L)**		**0**	**5**	**15**	**20**	**25**	**30**	**35**	**40**		
**Free acetaldehyde concentration (mg/L)**	**S1_OT**	0.0 ± 0.12	0.6 ± 0.09	2.5 ± 0.72	4.6 ± 0.22	6.5 ± 0.24	9.6 ± 0.68	13.1 ± 1.28	21.1 ± 2.06		
	**S2_OT**	0.0 ± 0.02	0.0 ± 0.03	0.0 ± 0.16	0.0 ± 0.25	0.0 ± 0.37	0.0 ± 0.12	0.0 ± 0.15	0.7 ± 0.05		
**B. Measured free acetaldehyde concentrations of spiked Syrah red wine samples for HCATA**											
**Acetaldehyde addition (mg/L)**		**0**	**5**	**15**	**25**	**40**	**55**	**70**	**85**	**100**	**120**
**Free acetaldehyde concentration (mg/L)**	**S1_HCATA**	0.0 ± 0.13	0.0 ± 0.9	5.1 ± 0.49	9.15 ± 0.22	21.3 ± 1.25	31.8 ± 3.1	41.9 ± 0.6	53.1 ± 5.2	63.6 ± 3.1	74.6 ± 1.1
	**S2_HCATA**	0.0 ± 0.03	0.0 ± 0.03	0.0 ± 0.07	0.0 ± 0.21	2.03 ± 0.14	8.85 ± 0.4	18.0 ± 0.03	32.05 ± 0.31	39.8 ± 0.3	52.4 ± 0.39

**Table 4:** Sensory thresholds for acetaldehyde for the Syrah red wine samples. Values represent means of triplicate determination ± standard deviation.

**Syrah 1**	**Acetaldehyde addition (mg/L)**		**5**	**15**	**20**	**25**	**30**	**35**	**Coeff a**	**Coeff b**	**OT (mg/L)**
	**Free Acetaldehyde concentration (mg/L)**		0.6	2.5	4.6	6.5	9.6	13.1			
	**% correct answers**	**Rep 1**	50%	33%	67%	42%	58%	50%	−0.14	0.023	6.1 ± 3.7
	**% correct answers**	**Rep 2**	17%	17%	50%	42%	67%	67%	−1.53	0.20	7.7 ± 3.7
**Syrah 2**	**Acetaldehyde addition (mg/L)**		**5**	**15**	**20**	**25**	**30**	**40**	**Coeff a**	**Coeff b**	**OT (mg/L)**
	**Free Acetaldehyde concentration (mg/L)**		0	0	0	0	0	0.7			
	**% correct answers**	**Rep 1**	17%	17%	50%	25%	25%	50%	/	/	/
	**% correct answers**	**Rep 2**	33%	50%	41%	33%	25%	42%	/	/	/

**Table 5:** Results of the Cochran’s Q test of each sample of Syrah red wine. *: Attribute whose citation frequency varies significantly between samples with α = 0.1; **: Attribute whose citation frequency varies significantly between samples with α = 0.05; ***: Attribute whose citation frequency varies significantly between samples with α = 0.01. Families–Sub-families–olfactory terms.

Attributes	*p*-Values Syrah 1	*p*-Values Syrah 2	Attributes	*p*-Values Syrah 1	*p*-Values Syrah 2
Fruity	0.488	0.04 **	Pastry	0.416	0.109
Red berries	0.016 **	0.221	Pastry	0.940	0.109
Strawberry	0.312	0.091 *	Vanilla	0.315	0.231
Blackberry	0.250	0.521	Cinnamon	0.437	0.050 *
Blackcurrant	0.216	0.514	Brioche	0.513	0.049 **
Stewed fruit	0.502	0.395	Pastry spices	0.124	0.587
Baked apple	0.0001 ***	0.056 *	Yeast	0.395	0.402
Jam	0.231	0.858	Malt	0.798	0.154
Overripe fruit	0.077 *	0.388	Animal	0.474	0.103
Overripe apples	0.007 **	0.347	Animal	0.740	0.064 *
Dried fruit	0.369	0.996	Leather	0.839	0.090 *
Dried figs	0.547	0.028 **	Meat juice	0.098 *	0.193
Floral	0.241	0.384	Lactic	0.317	0.752
Fresh flowers	0.087 *	0.980	Lactic	0.317	0.698
Dried flowers	0.700	0.884	Fresh butter	0.298	0.353
Vegetal	0.078 *	0.070 *	Empyreumatic	0.861	0.592
Fresh plants	0.057 *	0.094 *	Empyreumatic	0.861	0.267
Fresh green apples	0.112	0.533	Cocoa	0.279	0.098 *
Dry plants	0.740	0.395	Burnt	0.185	0.109
Undergrowth	0.388	0.822	Caramel	0.151	0.788
Spicy	0.285	0.738	Chemical	0.815	0.116
Spicy	0.365	0.462	Amylic	0.131	0.678
Licorice	0.141	0.320	Chemical	0.324	0.232
Aromatic plants	0.022 **	0.415	Nail polish remover	0.204	0.677
Mint	0.255	0.234	Oxidized	0.940	0.173
			Oxidized green apple	0.746	0.009 ***
			Sulfur	0.925	0.276

## Data Availability

Data are contained within the article and Supplementary Material.

## Supplementary Materials

The following supporting information can be downloaded at: https://www.mdpi.com/article/10.3390/foods11121693/s1, Table S1: Olfactory standards for panel training; Table S2: Detailed concentration of monomeric anthocyanins. Concentrations are expressed in mg.L^−1^ of Malvidin-3-O-glucoside equivalent. Values represent means of triplicate determination ± standard deviation; Table S3: Detailed concentration of flavanols after phloroglucinolysis. Concentrations are expressed in g.L^−1^ of each compound estimated using their response factors relative to catechin. Values represent means of triplicate determination ± standard deviation; Table S4: Reproducibility index and panel performance.
