# Supplementary material for: Impact of Acetaldehyde Addition on the Sensory Perception of Syrah Red Wines
Source: Foods. 2022 Jun 9;11(12):1693. doi: 10.3390/foods11121693 (PMC9223084; doi:10.3390/foods11121693)
Supplement: Supplementary file 1 [file foods-11-01693-s001.zip › foods-1754065-supplementary.pdf]

SUPPLEMENTARY INFORMATION

Impact of acetaldehyde addition on sensory perception of Syrah red wines

Luca Garcia, Cédrine Perrin, Valérie Nolleau, Teddy Godet, Vincent Farines, François Garcia, Soline Caillé and Cédric Saucier

Table of contents

	Page
**Table S1.** Olfactory standards for panel training	S-2
**Table S2**. Detailed concentration of monomeric anthocyanins. Concentrations are expressed in mg.L^{-1} of Malvidin-3-O-glucoside equivalent. Values represent means of triplicate determination ± Standard Deviation	S-4
**Table S3.** Detailed concentration of flavanols after phloroglucinolysis. Concentrations are expressed in g.L^{-1} of each compound estimated using their response factors relative to catechin. Values represent means of triplicate determination ± Standard Deviation	S-4
**Table S4.** Reproducibility index and panel performance.	S-5

Table S1: Olfactory standards for panel training.

Family	Subfamily	Descriptors	product added to a syrah wine for the realisation of olfactory standards
**Fruity**	Red berries	Strawberry	crushed strawberries
		Blackcurrant	blackcurrant berries, blackcurrant liquor
		Blackberry	blackberry berries, blackberry liquor
		Raspberry	raspberry berries, raspberry liquor
	Stewed fruit	Prune	prune juice
		Jam	red fruit jam
		Baked apple	baked apple pieces
	Dry fruit	Coconut	wiskey lactone
		Hazelnut	hazelnut powder
		Nut	nut powder
		Dried Fig	pieces of dried figs
	Overripe fruit	Overripe apples	overripe apples pieces
**Floral**	Fresh flowers	Violet	alpha-ionone solution
		White flowers	linalol solution
		Rose	rose hydrolate
	Dried flowers	Faded roses	rose petal powder
**Vegetal**	Fresh plant	Grass	fresh cut grass
		Fresh green apples	green apple, granny variety
		Peppers	pieces of peppers green and red
	Dry plant	Tobacco	blond tobacco
		Black tea	black tea
	Undergrowth	Humus	humus
		Truffle	white truffle vinegar
		Mushroom	2 octanol solution, piece of mushroom
**Spicy**	Spicy	Licorice	licorice candy
		Clove	clove powder
		Black pepper	black pepper
		Nutmeg	nutmeg powder
	Aromatic plants	Thyme	thyme powder
		Laurel	laurel leaf
		Eucalyptus	eucalyptus essential oil
		Black olive	pieces of black olives
		Mint	mint leaves
		Anise	green aniseed powder
**Pastry**	Pastry	Vanilla	vanilla powder
		Cinnamon	cinnamon powder

		Brioche	pieces of brioche
		Biscuit	piece of butter biscuit
		Pastry spices	4-spice mixture
		Praline	praline splinters
	Yeast	Malt	brewer's yeast
**Animal**	Animal	Leather	éthyl-4-phenol solution
		Meat juice	2 methyl 3 furanthiol solution
**Lactic**	Lactic	Fresh butter	melted butter
		Rancid butter	melted rancid butter
		Milk	condensed milk, fermented milk
**Empyreumatic**	Empyreumatic	Cocoa	cocoa powder
		Chocolate	dark chocolate chips
		Coffee	coffee powder
		Smoked, Burnt	smoked food flavouring
		Toasted bread	pieces of toasted bread
		Caramel	liquid caramel
**Chemical**	Amylic	Candy	isoamyl acetate solution
		Banana	banana candy
	Chemical	Nail polish remover	nail polish remover
		Varnish	isoamyl acetate solution
	Oxidized	Oxidized green apple	green apple cut and left to air for 24 hours
		Sweet wine	sweet grenache wine
	Sulfur	Tar	Heated tar
		Sulphur	burnt sulphur wick

Table S2. Detailed concentration of monomeric anthocyanins. Concentrations are

Judges	Syrah 1		Syrah 2		Judge performance
	Number of terms listed	Ri	Number of terms listed	Ri	
n°1	70	0.27	50	0.30	**
n°2	93	0.37	85	0.50	***
n°3	47	0.30	51	0.13	*
n°4	61	0.20	76	0.14	*
n°5	78	0.27	70	0.26	**
n°6	87	0.46	81	0.39	***
n°7	80	0.53	81	0.34	***
n°8	57	0.37	59	0.28	**
n°9	79	0.26	77	0.42	***
n°10	81	0.41	77	0.43	***
n°12	70	0.16	60	0.28	*
n°13	18	0.31	19	0.25	*

expressed in $mg.L^{-1}$ of Malvidin-3-O-glucoside equivalent. Values represent means of triplicate determination ± Standard Deviation.

Table S3 Detailed concentration of flavanols after phloroglucinolysis. Concentrations are expressed in $g.L^{-1}$ of each compound compound estimated using their response factors relative to catechin. Values represent means of triplicate determination ± Standard Deviation.

**S1**	**Delphidin 3 O Glu**	**Cyanidin 3 O Glu**	**Petunidin 3 O Glu**	**Peonidin 3 O Glu**	**Malvidin 3 O Glu**
	7.65 ± 0.04	1.06 ± 0.06	10.60 ± 0.38	19.68 ± 0.15	108.35 ± 0.38
	**Delphidin 3 O acetyl Glu**	**Cyanidin 3 O acetyl Glu**	**Petunidin 3 O acetyl Glu**	**Peonidin 3 O acetyl Glu**	**Malvidin 3 O acetyl Glu**
	1.26 ± 0.15	0.18 ± 0.03	8.54 ± 0.07	8.56 ± 0.06	30.29 ± 0.17
	**Cyanidin 3 O coum Glu**	**Petunidin 3 O coum Glu**	**Peonidin 3 O coum Glu**	**Malvidin 3 O coum Glu**	
	0.44 ± 0.04	1.39 ± 0.3	5.47 ± 0.03	19.74 ± 0.23	
**S2**	**Delphidin 3 O Glu**	**Cyanidin 3 O Glu**	**Petunidin 3 O Glu**	**Peonidin 3 O Glu**	**Malvidin 3 O Glu**
	20.39 ± 0.84	3.54 ± 0.2	29.60 ± 0.18	59.47 ± 0.25	231.14 ± 0.51
	**Delphidin 3 O acetyl Glu**	**Cyanidin 3 O acetyl Glu**	**Petunidin 3 O acetyl Glu**	**Peonidin 3 O acetyl Glu**	**Malvidin 3 O acetyl Glu**
	3.44 ± 0.25	0.79 ± 0.21	19.09 ± 0.13	21.09 ± 0.1	71.02 ± 0.07
	**Cyanidin 3 O coum Glu**	**Petunidin 3 O coum Glu**	**Peonidin 3 O coum Glu**	**Malvidin 3 O coum Glu**	
	2.18 ± 0.1	5.28 ± 0.5	14.32 ± 0.08	31.26 ± 0.13	

Table S4: Reproducibility index and panel performance**.**

	Gallocatechin-phloroglucinol	Catechin-phloroglucinol	Epicatechin-phloroglucinol	Catechin	Epicatechin gallate-phloroglucinol	Epicatechin	Gallocatechin	Epicatechin gallate	Total flavanols
**S1**	0.19 ± 0.02	0.02 ± 0.008	0.10 ± 0.01	0.07 ± 0.003	0.11 ± 0.01	0.04 ± 0.001	0.12 ± 0.01	0.04 ± 0.001	0.69 ±0.04
**S2**	0.21 ± 0.01	0.03 ± 0.006	0.35 ± 0.02	0.12 ± 0.002	0.12 ± 0.015	0.08 ± 0.003	0.27 ± 0.03	0.09 ± 0.002	1.28 ± 0.05

***: very performant judges (a lot of cited and repeated terms); **: judges with a rather good overall performance; *: less performant judges
